# Highly Selective CO_2_ Reduction to Pure Formic Acid Using a Nafion‐TiO_2_ Composite Porous Solid Electrolyte

**DOI:** 10.1002/advs.202515967

**Published:** 2025-10-13

**Authors:** Yeomin Kang, Kooyoung Jung, Jusung Kim, Ki Tae Park

**Affiliations:** ^1^ Department of Chemical Engineering Konkuk University 120 Neungdong‐ro, Gwangjin‐gu Seoul 05029 Republic of Korea

**Keywords:** CO_2_ reduction, electrocatalysis, porous solid electrolyte, solid‐state electrolyte, TiO_2_

## Abstract

The electrochemical reduction of CO_2_ (CO_2_RR) to liquid fuels and chemicals offers a sustainable route to close the carbon cycle. Solid‐state electrolyte (SSE) systems have recently emerged as a breakthrough platform, enabling the direct synthesis of high‐purity liquid products. Here, a novel Nafion‐based porous solid electrolyte (NPSE) with tunable thickness and porosity is presented, replacing conventional bead‐type ion‐exchange resins. The structural tunability of NPSEs plays a critical role in determining CO_2_RR performance. Furthermore, a Nafion‐titanium dioxide (TiO_2_) composite NPSE (NPSE‐TiO_2_) is developed, in which TiO_2_ nanoparticles significantly enhance the selectivity and productivity of formic acid. The NPSE‐TiO_2_ achieves a record‐high Faradaic efficiency (FE_HCOOH_) of 98.6%, the highest value reported to date among SSE systems, along with a high partial current density (*j*_HCOOH_) of 431.8 mA cm^−2^, yielding electrolyte‐free formic acid with a single‐pass concentration of 17.4 wt.%. Mechanistic investigations reveal that TiO_2_ promotes the transport of formate anions (HCOO^−^), suppressing back‐diffusion of formic acid to the cathode and stabilizing cathodic pH.

## Graphical abstract

A Nafion‐based porous solid electrolyte (NPSE) is reported, optimized by thickness, porosity, and titanium dioxide (TiO_2_) incorporation for selective electrochemical reduction of CO_2_ to formic acid. The NPSE–TiO_2_ composite achieves a FE_HCOOH_ of 98.6%, *j*_HCOOH_ of 431.8 mA cm^−2^, and single‐pass concentration of 17.4 wt.%. Mechanistic insights reveal that TiO_2_ enhances formate transport, suppressing back‐diffusion of formic acid and stabilizing cathodic pH.

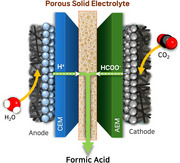

## 1 Introduction

The increasing atmospheric concentration of CO_2_ due to fossil fuel combustion and industrial activities has heightened the urgency of developing carbon capture and utilization (CCU) technologies.^[^ ^1^ ^]^ Among various electrochemical CO_2_ reduction reaction (CO_2_RR) products, formic acid (HCOOH) is particularly attractive due to its high energy density, liquid‐phase storage capability, and use as both a hydrogen carrier and a key industrial chemical. Compared to gaseous products such as carbon monoxide, formic acid offers distinct advantages in handling, storage, and transportation due to its liquid phase at ambient conditions, making it a highly promising candidate for sustainable energy and chemical production. However, a major challenge in formic acid production via CO_2_RR is that the reaction often takes place in alkaline or neutral conditions, leading to the formation of formate salts mixed with electrolyte rather than free formic acid.^[^ ^2^, ^3^, ^4^, ^5^, ^6^, ^7^, ^8^, ^9^, ^10^, ^11^, ^12^, ^13^, ^14^, ^15^, ^16^ ^]^ This necessitates additional downstream purification and acidification steps, increasing process complexity and energy requirements.^[^ ^17^, ^18^ ^]^ To address this challenge, Yang et al. introduced a solid‐state electrolyte (SSE) system for a CO_2_RR electrolyser, which consisted of three distinct compartments separated by an anion exchange membrane and a cation exchange membrane. This configuration enabled the direct production of high‐purity formic acid; however, it suffered from relatively low product selectivity and current density.^[^ ^19^ ^]^ Since then, various research groups have developed increasingly advanced catalyst designs to improve the Faradaic efficiency and current density of CO_2_RR.^[^ ^20^, ^21^, ^22^, ^23^, ^24^, ^25^, ^26^, ^27^ ^]^ In addition, the SSE system design has been expanded to the production of other value‐added products. For example, Wi et al. demonstrated that the SSE cell configuration enables the production of C_2_ products,^[^ ^28^ ^]^ while Xia et al. and Zhang et al. reported promising results for oxygen reduction for hydrogen peroxide production.^[^ ^29^, ^30^ ^]^ Additionally, Liu et al. have incorporated SSE cell configurations in the particularly difficult electrochemical synthesis of urea.^[^ ^31^ ^]^ Despite these advances, the solid electrolyte system still suffers from high overall cell voltages, reducing energy efficiency compared to conventional electrochemical setups.^[^ ^18^, ^32^ ^]^

While most studies focus on catalyst development to improve selectivity and activity, the design and operation of the electrolysis cell themselves play equally crucial roles in achieving efficient CO_2_RR. Among the various cell components, SSE materials have been identified as a major source of internal resistance, leading to high cell voltages.^[^ ^22^ ^]^ Optimizing the architecture and physicochemical properties of SSEs may provide a more cost‐effective and scalable strategy for reducing energy losses and improving overall process efficiency. Recently, Cherniack et al. introduced an ionomer wafer to further investigate the interfacial phenomena between components of the SSE cell.^[^ ^33^ ^]^ However, this design exhibited low selectivity toward CO_2_RR products due to insufficient anion transport and back‐diffusion of produced formic acid to the cathode, leading to product loss from the center compartment and a decrease in selectivity of CO_2_RR due to local acidification, which promotes the hydrogen evolution reaction (HER).

Herein, we report a Nafion‐based porous solid electrolyte (NPSE) as an advanced SSE platform for CO_2_RR to formic acid. Through systematic optimization of the key parameters of NPSE, including thickness, porosity, and chemical composition, it was successfully verified that the NPSE design improvements can be as impactful as catalyst innovations, offering a practical and cost‐effective pathway for advancing CO_2_RR technologies. Beyond structural tuning, we further explore the incorporation of titanium dioxide (TiO_2_) nanoparticles into the NPSE matrix as a strategy to enhance ionic properties and address limitations associated with formate anion transport and back‐diffusion of formic acid. This study highlights the importance of solid electrolyte engineering in advancing SSE systems capable of high selectivity, improved energy efficiency, and scalable production of high‐purity formic acid.

## 2 Results and Discussion

**Figure 1:**
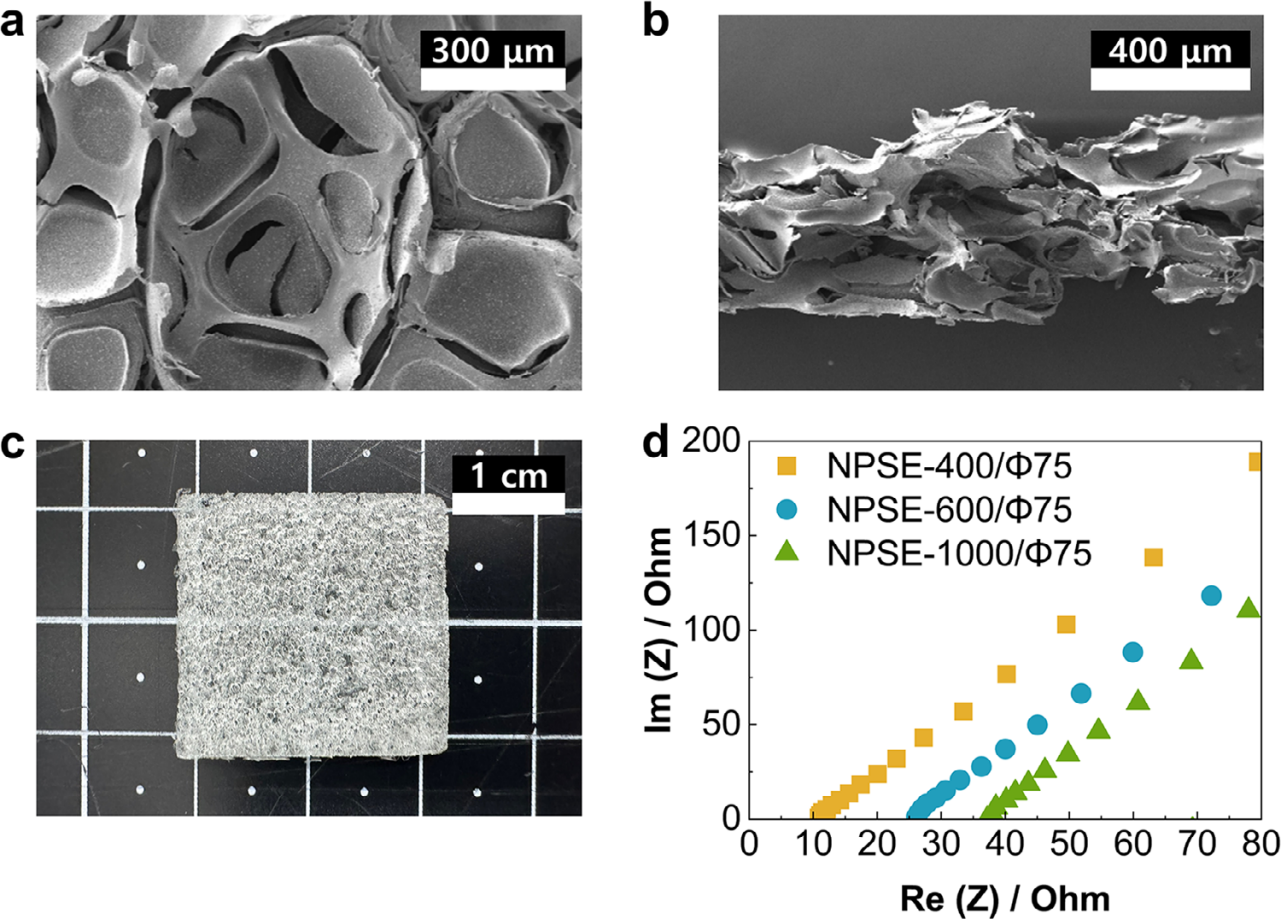
Morphological and electrochemical characterization of NPSE samples. a) Top‐view and b) cross‐sectional SEM images of NPSE‐600/Φ75, revealing a well‐defined porous structure. c) Digital photograph of NPSE‐600/Φ75. d) Nyquist impedance plots (1 MHz to 100 Hz, E_OC_, 10 mV amplitude) for isolated NPSE samples according to thickness.

NPSEs were prepared via a metal foam leaching method, as shown in Figure S1 (Supporting Information). The prepared NPSEs were a continuous mesh of ionomers, forming a translucent, sponge‐like gel having regular pores of ≈500 µm (**Figure** **1**a–c). Various strategies have been explored to introduce porosity into Nafion membranes, including the use of volatile porogenes^[^ ^34^, ^35^ ^]^ and salting out techniques.^[^ ^36^, ^37^ ^]^ However, these conventional methods often fail to produce open‐pore structures capable of supporting sufficient liquid flow, an essential requirement for the efficient recovery of liquid products in SSE systems. In contrast, our metal foam leaching method enables the reliable fabrication of porous solid electrolytes with well‐defined open‐pore architectures that facilitate effective and stable liquid transport throughout the solid electrolyte matrix.

The proposed method also enables precise control over key structural parameters of the NPSE, including thickness and porosity. The thickness was adjusted by varying the amount of ionomer dispersion injected, in combination with custom‐fabricated polydimethylsiloxane (PDMS) wells and nickel foams (NF) with matched depth and thickness. NPSEs with target thicknesses of 400, 600, and 1000 µm were successfully fabricated (Figure S2, Supporting Information). Likewise, by tuning the porosity of the metal foam template, the porosity of the resulting NPSEs was adjusted to 75%, 60% and 50%, respectively (Figure S3, Supporting Information). With denser NFs, the resulting NPSEs had a closer‐knit mesh, leading to a lower porosity solid electrolyte. The prepared NPSE samples were denoted as NPSE‐thickness/porosity based on their thickness and porosity. For example, NPSE‐600/Φ60 refers to a NPSE with a thickness of 600 µm and a porosity of 60%. A summary of the NPSE formulations and their structural characteristics is presented in Table S1 (Supporting Information). Further details on the preparation procedure are available in the Experimental Section of Supporting Information.

NPSE samples were characterized to elucidate their structural and compositional properties. As shown in Figure S4 (Supporting Information), scanning electron microscopy (SEM) for high‐resolution imaging with energy‐dispersive X‐ray spectroscopy (EDS) revealed that the NPSE matrix was primarily composed of carbon, fluorene, and sulfur, consistent with the expected chemical composition of Nafion. Negligible amounts of nickel were detected by EDS (Table S2, Supporting Information), indicating that the dilute piranha solution used was effective at removing the NF scaffold, while preserving the porous structure of the solid polymer electrolyte.

To evaluate the ion‐conductive properties of the NPSEs, electrochemical impedance spectroscopy (EIS) measurements were carried out using a custom‐designed solid electrolyte analysis cell (Figure S5, Supporting Information). As shown in Figure 1d, the solution resistance (R_S_) increased with NPSE thickness, indicating higher proton transfer resistance due to the extended diffusion path. Notably, the R_S_ of NPSE‐600/Φ60 was nearly identical to that of NPSE‐400/Φ75 (Figure S6, Supporting Information), suggesting that both reducing thickness and lowering porosity are effective strategies for minimizing the ionic resistance of NPSEs.

In addition, the ion exchange capacity (IEC) of the NPSEs was measured via back titration (Figure S7, Supporting Information), with resulting IEC values of ≈0.9 meq mL^−1^, which are consistent with the reported IEC of Nafion (0.83–1.02 meq g^−1^).^[^ ^38^ ^]^ These results, together with the aforementioned EDS analysis, confirm that the leaching process does not chemically degrade the Nafion ionomer, while effectively and completely removing the nickel foam scaffold.

**Figure 2:**
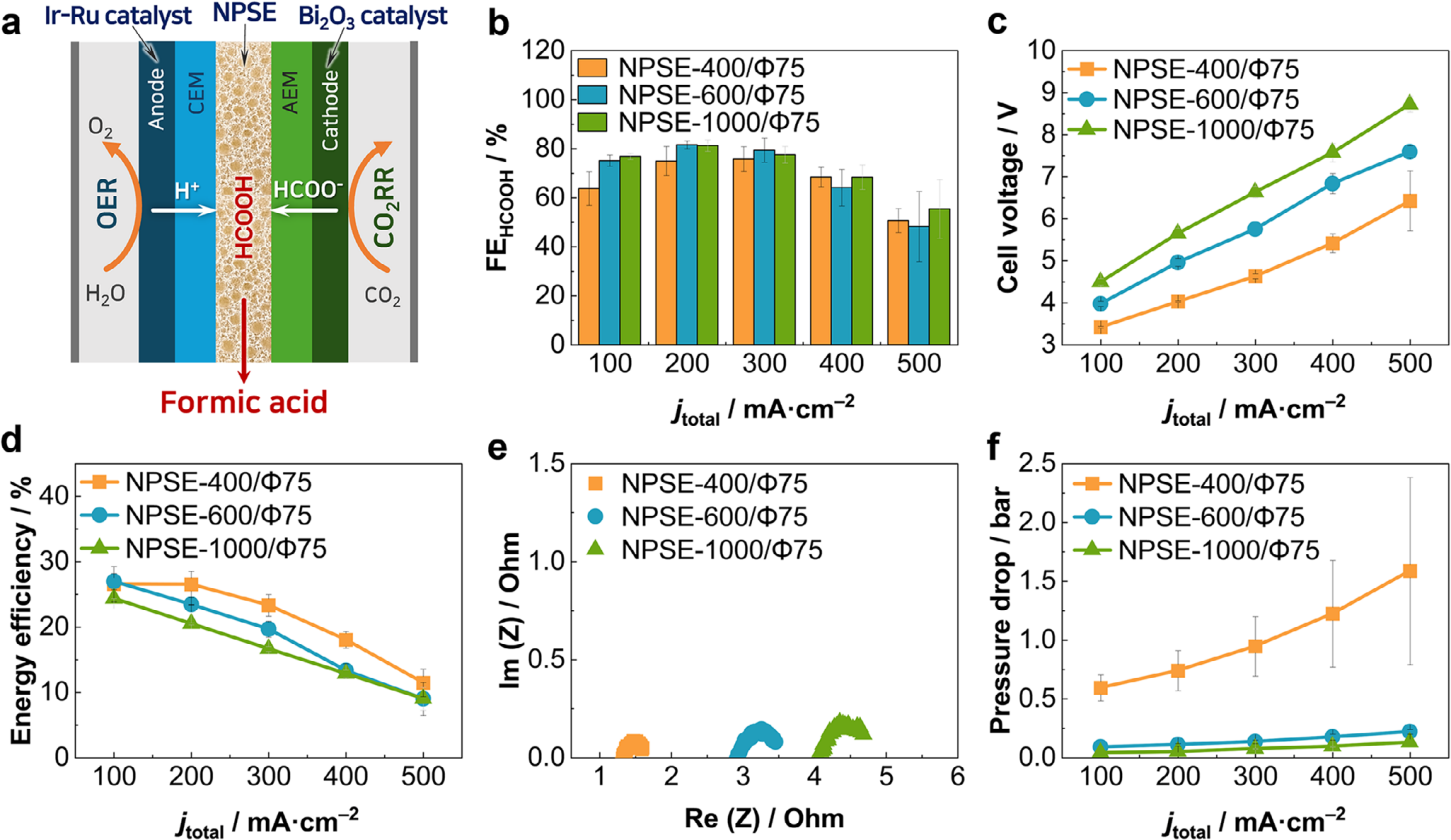
a) Schematic illustration of the solid‐state electrolyte (SSE) cell employing Nafion‐based porous solid electrolyte (NPSE). Performance metrics for CO_2_RR as a function of NPSE thickness: b) Faradaic Efficiency (FE) for formic acid, c) full cell voltage, d) energy efficiency, e) Nyquist impedance plots (1 MHz to 10 Hz, 3.8V_cell_, 30 mV amplitude), and f) pressure drop across the center compartment.

To evaluate the CO_2_RR performance, the fabricated NPSEs were introduced into the center compartment of solid‐state electrolyte (SSE) cells, where the cathodic catalyst was commercially available bismuth oxide (Bi_2_O_3_) nanoparticles and the anode catalyst was a mix of iridium (Ir) and ruthenium (Ru) (**Figure** **2**a). The cells were operated in an experimental setup as shown in Figure S8 (Supporting Information). CO_2_RR performances were monitored using the chronopotentiometry (CP) technique, while electrochemical characteristics of the system were analyzed via EIS and linear sweep voltammetry (LSV). The structural parameters of the NPSEs and their correlation with CO_2_ reduction performance were systematically investigated.

As the NPSE thickness increased from 400 to 1000 µm at a fixed porosity of 75%, the Faradaic efficiency (FE) for formic acid generally improved (Figure 2b). Among the tested samples, NPSE‐600/Φ75 achieved the highest FE of 81.6% at a current density of 200 mA cm^−2^ (Figure 2b). Notably, the thickest sample, NPSE‐1000/Φ75, demonstrated better selectivity at higher current densities above 400 mA cm^−2^ compared to that of thinner NPSEs. This enhancement in selectivity is attributed to the increased distance between the cathode and the acidic anode with thicker NPSE, which increases the local pH near the cathode and consequently suppresses the competing HER. Despite the improved CO_2_RR selectivity observed with NPSE‐1000/Φ75, the substantially higher cell voltage and resulting energy demand rendered the use of thicker NPSEs impractical for efficient CO_2_RR, as shown in Figure 2c. A clear correlation was observed between NPSE thickness and cell voltage, with NPSE‐1000/Φ75 exhibiting a high cell voltage of 8.72 V at 500 mA cm^−2^.

When energy efficiency (EE), which accounts for both Faradaic efficiency (FE) and cell voltage, was used as a performance metric, NPSE‐400/Φ75 consistently outperformed the thicker NPSEs, as shown in Figure 2d. This improvement is primarily attributed to reduced proton transport resistance in thinner NPSEs. Nyquist plots (Figure 2e) further support this finding, revealing a substantial decrease in R_s_ and a moderate reduction in charge transfer resistance (R_CT_) with decreasing NPSE thickness. Additionally, linear sweep voltammetry (LSV) results (Figure S9a, Supporting Information) showed that current density inversely correlated with NPSE thickness, supporting the trend of enhanced ionic conductivity and reduced resistance in thinner NPSE configurations.

However, although thinner NPSEs exhibited enhanced energy efficiency for formic acid production, excessive pressure drop was observed for NPSE‐400/Φ75, especially at high current densities. At 500 mA cm^−2^, the pressure drops across the center compartment reached ≈1.59 bar, as represented in Figure 2f. This significant increase of pressure drop with current density observed in NPSE‐400/Φ75 is primarily attributed to the generation of CO_2_ gas at increased current density.^[^ ^19^ ^]^ Upon applying current to the SSE cell, hydronium ions (H_3_O^+^) are migrated from the anode, while a mixture of formate and carbonate/bicarbonate anions is delivered from the cathode into the center compartment through the cation exchange membrane (CEM) and anion exchange membrane (AEM), respectively. Within the acidic environment of the center compartment, the carbonate and bicarbonate species are rapidly converted to CO_2_ gas, leading to volumetric expansion and a sharp increase in internal pressure. This excessive pressure drop compromised the operational stability of NPSE‐400/Φ75, making it unsuitable for practical applications.

Interestingly, the outlet concentration of formic acid remained nearly constant across NPSEs of varying thickness (Figure S9b, Supporting Information). This behavior is attributed to a trade‐off between pressure drop‐induced flow resistance and residence time. In thinner NPSEs, although partial current densities were lower, the elevated pressure drop slowed liquid flow rate and increased residence time, resulting in comparable product concentrations. Considering these factors, particularly the severity of pressure‐related limitations in thinner membranes and relatively low Faradaic efficiency, the 600 µm NPSE was selected as the minimum viable configuration for further investigation.

**Figure 3:**
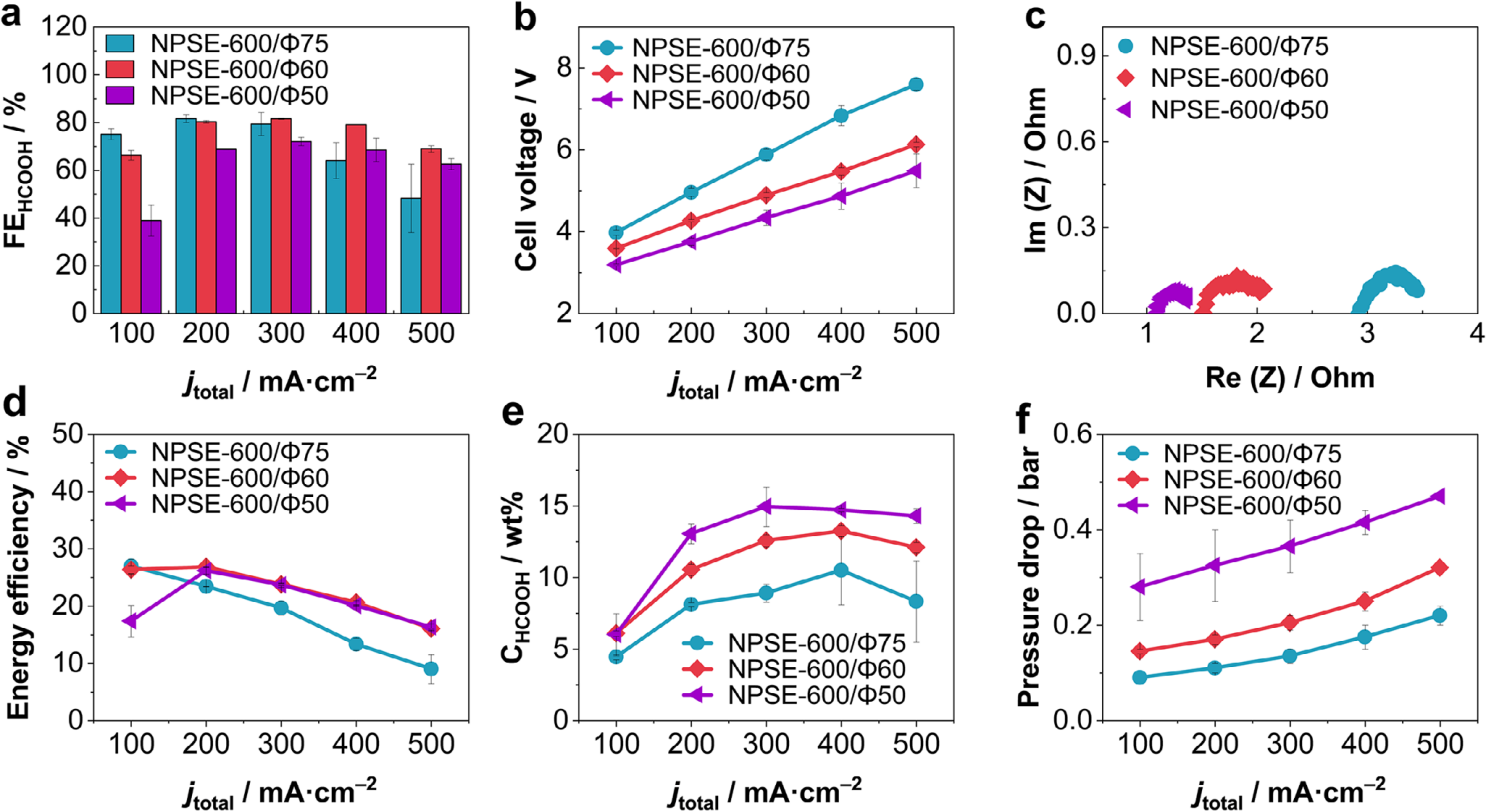
CO_2_RR performance metrics as a function of NPSE porosity. a) Faradaic efficiency for formic acid (FE_HCOOH_), b) Cell voltage, c) Nyquist impedance plots (1 MHz to 10 Hz, 3.8 V_cell_, 30 mV amplitude), d) Energy efficiency (EE), e) Outlet concentrations of formic acid, and f) Pressure drop across the center compartment.

To investigate the influence of the porosity of NPSE on CO_2_RR performance, a series of NPSEs with different porosities (Φ75, Φ60, and Φ50) but fixed thickness (600 µm) was evaluated in an SSE cell. As shown in **Figure** **3**a, reducing the porosity from 75% to 60% led to significantly higher FE_HCOOH_ at higher current densities, with NPSE‐600/Φ60 achieving 69.0% FE_HCOOH_ at 500 mA cm^−2^, 22%p higher than NPSE‐600/Φ75. However, when the porosity was further decreased to 50%, the FE_HCOOH_ dropped significantly compared to NPSE‐600/Φ60. These results suggest that lowering porosity can be beneficial for reducing cell voltages at a given current density (Figure 3b), but overly dense NPSEs facilitate excessive proton transport at the NPSE/cathode interface, thereby accelerating HER.

The corresponding cell voltages presented in Figure 3b exhibit a clear trend of decreasing with reduced porosity at all current densities. This indicates a proportional decrease in internal resistance due to the denser structure of NPSE, which promotes ion transport. The EIS spectra (Figure 3c) and LSV curves (Figure S10, Supporting Information) further support this observation, showing decreasing ohmic resistance with increasing NPSE density.

Energy efficiency results are summarized in Figure 3d, where NPSE‐600/Φ60 and NPSE‐600/Φ50 clearly outperform the Φ75 sample across the entire tested current range, particularly at higher current densities. Except at 100 mA cm^−2^, the NPSE‐600/Φ60 and NPSE‐600/Φ50 show comparable energy efficiency, as the decrease in cell voltage of the NPSE‐600/Φ50 roughly offsetting the decrease in FE_HCOOH_. These results highlight the importance of structural optimization in minimizing energy losses, an essential consideration for the practical scale‐up of CO_2_ electrolyzers.

The decrease in NPSE porosity led to an increase in the outlet concentration of formic acid (Figure 3e), with only a marginal increase in pressure drop of 0.07 and 0.24 bar for NPSE‐600/Φ60 and NPSE‐600/Φ50, respectively, compared to NPSE‐600/Φ75 (Figure 3f). Unlike the case of varying NPSE thickness (Figure S9b, Supporting Information), this result is particularly notable because product concentration strongly impacts the economic viability of CO2RR, owing to the high costs of downstream separation and purification.^[^ ^17^, ^20^, ^25^ ^]^ This correlation arises because decreasing NPSE porosity reduces internal void volume, thereby concentrating the generated formic acid within the transport pathways and leading to higher outlet product concentrations.

Interestingly, the cell resistance and corresponding operating cell voltage of NPSE‐600/Φ60 were nearly identical to those of NPSE‐400/Φ75. The only notable difference in CO_2_RR performance between the two NPSEs was the pressure drops across the center compartment. The NPSE‐600/Φ60 exhibited a significantly lower and more manageable maximum pressure drop of 0.32 bar (Figure 3f), compared to 1.59 bar for NPSE‐400/Φ75, enabling more stable operation. These results suggest that optimizing porosity, rather than simply reducing thickness, is a more effective strategy for enhancing proton transport with maintaining hydrodynamic and operational stability. Hence, NPSEs were optimized to a thickness of 600 µm and porosity of 60%.

**Figure 4:**
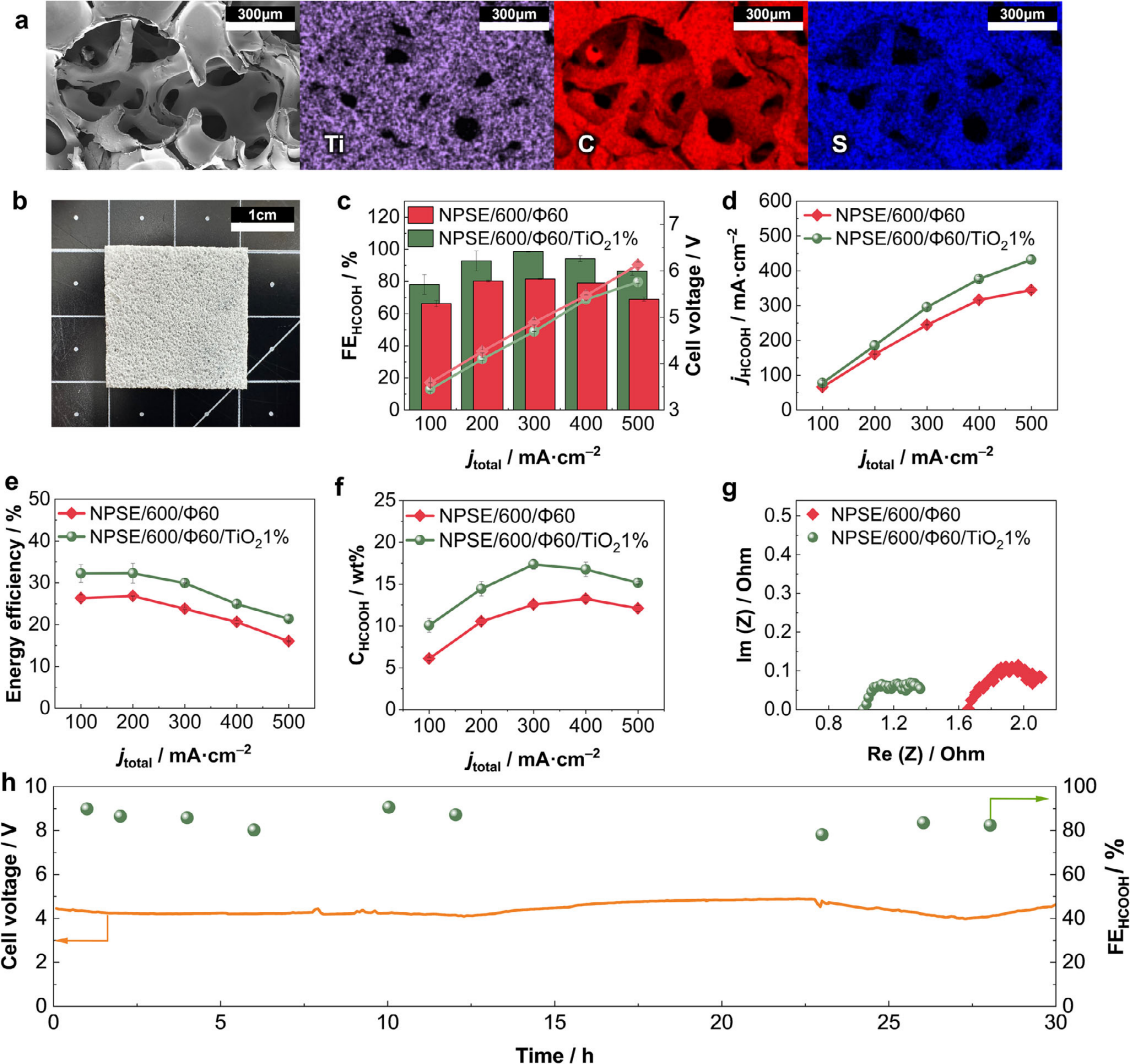
Characterization and CO_2_RR performance of NPSE‐600/Φ60/TiO_2_ composite electrolytes. a) SEM‐EDS mapping images of NPSE‐600/Φ60/TiO_2_ 1%, showing uniform dispersion of Ti within the Nafion matrix (C and S). b) Digital photograph of NPSE‐600/Φ60/TiO_2_ 1% sample. c) Faradaic efficiency for formic acid (FE_HCOOH_), d) Partial current density for formic acid production (*j*_HCOOH_), e) Energy efficiency, f) Outlet concentrations of formic acid, g) Nyquist impedance plots (1 MHz to 10 Hz, 3.8 V, 30 mV amplitude), and h) Long‐term stablity test results using NPSE‐600/Φ60/TiO_2_ 1% operating at 200 mA cm^−2^ over 30 h.

Following the identification of favorable structural parameters for NPSEs, CO_2_RR performance was further enhanced by the incorporation of TiO_2_ nanoparticles (TiO_2_ NPs) into the NPSE matrix. As an amphoteric oxide, TiO_2_ is known to function as both a Lewis and Brønsted acid.^[^ ^39^, ^40^ ^]^ Thus, its incorporation would increase the IEC of the solid electrolyte and further reduce ionic resistance. Uniform dispersion of TiO_2_ NPs within the NPSE was achieved by blending TiO_2_ slurry with Nafion dispersion with varying weight ratios (summarized in Table S3, Supporting Information). Successful incorporation was confirmed by SEM‐EDS analysis (**Figure** **4**a; Figures S11 and S12, Supporting Information), which also indicated complete removal of the Ni scaffold (Table S4, Supporting Information). All NPSE‐TiO_2_ composite samples were fabricated with a fixed thickness of 600 µm and a porosity of 60% (Figure S13, Supporting Information). The resulting NPSE‐600/Φ60/TiO_2_ maintained a similar morphology to unmodified NPSEs, except for a distinct opaque white coloration, as shown in Figure 4b.

The incorporation of TiO_2_ into NPSE‐600/Φ60 led to a notable enhancement in CO_2_RR performance. As shown in Figure 4c, the FE_HCOOH_ reached 98.6% at 300 mA cm^−2^, with a notably high selectivity of 86.4% maintained even at 500 mA cm^−2^. This corresponded to a maximum partial current density of 431.8 mA cm^−2^ for formic acid production (Figure 4d). Additionally, TiO_2_ incorporation also contributed to a reduction in cell voltage from 6.1 to 5.7 V at 500 mA cm^−2^ (Figure 4c). The synergetic effects of improved selectivity and reduced resistance led to a substantial improvement in energy efficiency and outlet concentration of formic acid, achieving values of 32.29% (Figure 4e) and 17.4 wt.% (Figure 4f), respectively.

EIS measurements (Figure 4g) showed that the incorporation of TiO_2_ NPs led to a reduction in solution resistance (R_s_), suggesting improved ionic transport. In addition, back titration measurements indicated up to a 75% increase in IEC compared to the unmodified NPSE‐600/Φ60 with increasing TiO_2_ loading (Figure s14, Supporting Information). However, EIS results from both the SSE cells and isolated NPSE samples (Figures S15d and S16, Supporting Information) exhibited increased R_s_ at higher TiO_2_ loadings. Moreover, the increase in IEC did not directly correlate with improvements in key CO_2_RR performance metrics such as FE_HCOOH_, cell voltage, *j*_HCOOH_, or energy efficiency as shown in Figure S15 (Supporting Information). Among the tested samples, NPSE‐600/Φ60/TiO_2_ 1% exhibited the highest CO_2_RR performance. This suggests that beyond an optimal TiO_2_ content, agglomeration of TiO_2_ particles creates non‐conductive domains that disrupt continuous ion transport pathways and increase overall resistance, thereby negating the benefits of enhanced IEC.

The operational stability of the NPSE‐600/Φ60/TiO_2_ 1% was evaluated over 30 h at a constant current density of 200 mA cm^−2^ (Figure 4h). The cell maintained high selectivity (FE_HCOOH_ > 80%) and a stable operating cell voltage (≈4 V) throughout the test. Given the well‐established mechanical and chemical robustness of Nafion and TiO_2_, the promising long‐term stability results are consistent with expectations.

**Figure 5:**
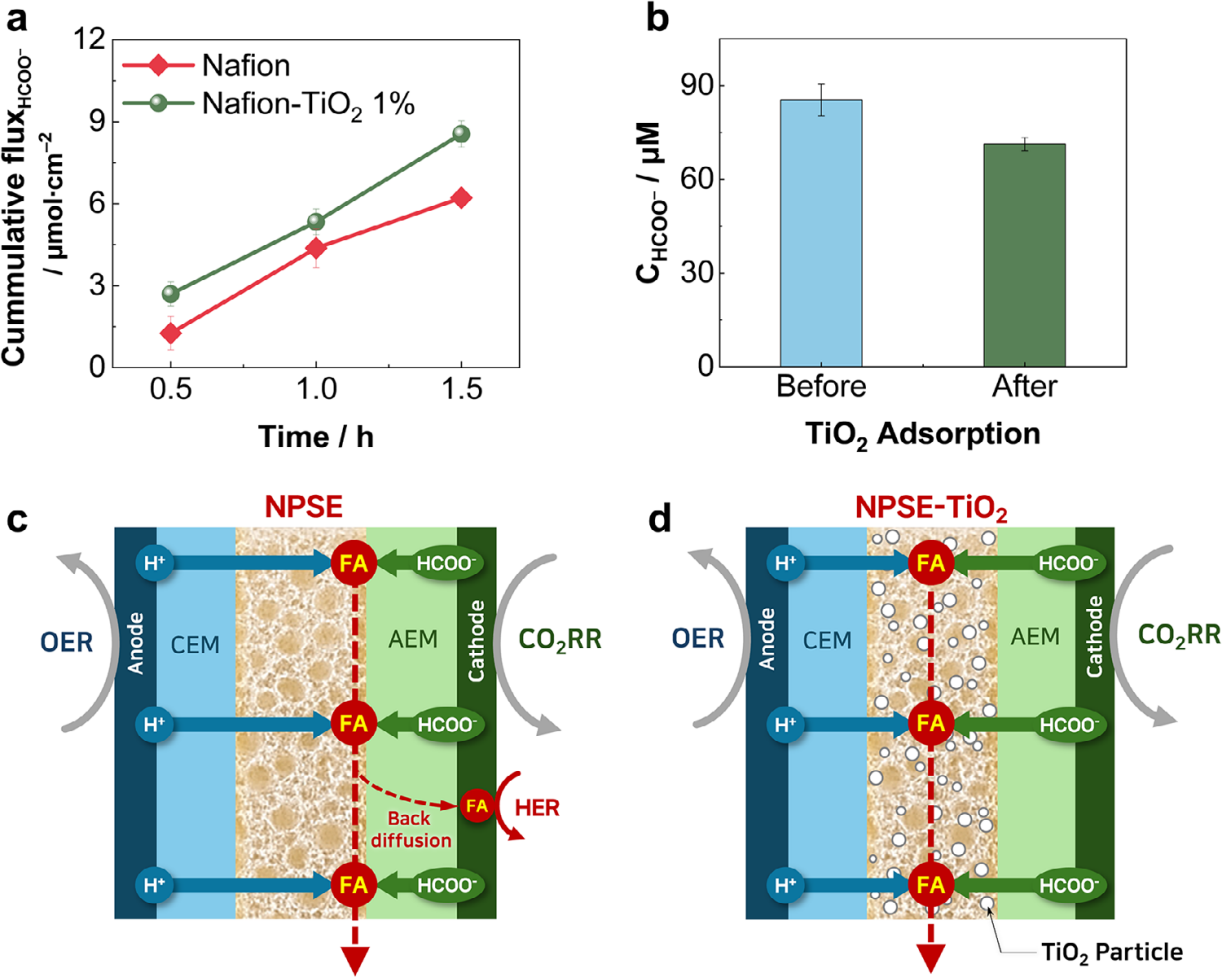
Mechanistic investigation of TiO_2_ nanoparticle function in NPSE. a) Cumulative formate ion (HCOO^−^) flux through either a pristine Nafion membrane or a Nafion‐TiO_2_ composite membrane. b) Formate concentrations of potassium formate solutions before and after TiO_2_ treatment. c,d) Schematic illustrations of SSE cell operation: (b) without and (c) with TiO_2_ nanoparticle incorporation.

However, the superior CO_2_RR performance for formic acid production, particularly the substantial increase in the FE_HCOOH_, achieved by NPSE‐600/Φ60/TiO_2_ 1%, cannot be fully attributed to the increase in IEC or the reduction in R_s_ alone. To further investigate whether TiO_2_ also affects the transport of formate anions (HCOO^−^) within the NPSE, diffusion experiments were conducted using both a pristine Nafion membrane and a Nafion‐TiO_2_ composite membrane containing 1 wt.% TiO_2_ NPs, as illustrated in Figure S17 (Supporting Information). Two chambers were separated by either a Nafion membrane or a Nafion‐TiO_2_ composite membrane, both of which were dense (non‐porous) and cast to have a nominal thickness of 50 µm. The donor chamber was filled with 0.5 m potassium formate (HCOOK) solution, while the receiving chamber contained deionized water. Formate concentrations in the receiving chamber were monitored periodically over 1.5 h, and the resulting cumulative flux of HCOO^−^ (µmol cm^−2^) through each membrane is presented in **Figure** **5**a.

Notably, the cumulative HCOO^−^ flux through the Nafion‐TiO_2_ composite membrane reached 8.56 µmol cm^−2^ after 1.5 h, a 37.6% increase of that compared to pristine Nafion membrane (6.22 µmol cm^−2^), as shown in Figure 5a. This result clearly indicates that the TiO_2_ incorporation enhances HCOO^−^ transport within the NPSE. To the best of our knowledge, this is the first reported evidence of TiO_2_ facilitating formate transport when embedded in an ion‐exchange membrane. Given that this enhancement was observed under purely diffusive conditions with a low concentration gradient (0.5 m), it is expected that HCOO^−^ transport would be even more pronounced under practical operating conditions with higher concentration differentials exceeding 10 wt.% (Figure 4f).

The mechanism by which TiO_2_ facilitates formate anions is proposed to be adsorption‐assisted transference. Formic acid is known to chemisorb on the surface of TiO_2_ in monodentate and bidentate configurations.^[^ ^41^ ^]^ To verify adsorption in the aqueous phase, a bulk adsorption experiment was conducted, in which a dilute solution of 85 µM HCOOK was treated with TiO_2_ nanoparticles. As shown in Figure 5b, the HCOOK solution showed significant decreases in formate concentration (C_HCOO_^−^) after 30 min. The density of surface adsorption site was calculated to be 5.43 µmol m^−2^ (Table S5, Supporting Information), consistent with reported values (5≈6 µmol m^−2^),^[^ ^41^ ^]^ thereby providing strong evidence for the adsorption‐assisted transference mechanism.

Crucially, the ability of the NPSE‐TiO_2_ composite to facilitate HCOO^−^ transport not only effectively suppresses the back‐diffusion of the produced formic acid but also improves the local pH environment at the cathode, both of which are critical for sustaining high CO_2_RR selectivity. Previous studies have pointed to local pH and back diffusion being a significant factor in the CO_2_RR selectivity of SSE systems.^[^ ^32^, ^42^, ^43^ ^]^ Since the NPSE is composed of a cation‐conductive Nafion ionomer, protons (H^+^) are readily transported through the NPSE, whereas formate anions (HCOO^−^) are less likely to be transported. Consequently, the recombination of H^+^ and HCOO^−^ to form formic acid predominantly occurs at the NPSE‐AEM interface near the cathode, as depicted in Figure 5b. In this configuration, the formic acid produced is susceptible to back‐diffusion toward the cathode, leading to product loss from the center compartment and a decrease in FE_HCOOH_ due to local acidification, which promotes the HER.

In contrast, in the NPSE‐TiO_2_ composite, the incorporation of TiO_2_ facilitates HCOO^−^ transport, thereby shifting the site of formic acid formation farther from the cathode. This spatial displacement effectively mitigates back‐diffusion of formic acid and sustains a more alkaline cathode environment, thereby enhancing CO_2_RR selectivity. Therefore, these findings demonstrate that the incorporation of TiO_2_ plays a crucial role in addressing key limitations of SSE cells, such as formic acid back‐diffusion and low CO_2_RR selectivity, by simultaneously enhancing IEC and anion transport, while stabilizing the local pH environment at the cathode.

## 3 Conclusion

In summary, we developed and systematically optimized Nafion‐based porous solid electrolytes (NPSEs) for solid‐state electrolyte (SSE) CO_2_RR systems for the selective production of pure formic acid. Structural variables such as thickness, porosity, and TiO_2_ incorporation were investigated to assess their impact on ion transport, selectivity, and energy efficiency. Our results show that NPSE thickness influences Faradaic efficiency, cell voltage, and energy efficiency, while porosity optimization further enhances system performance. Critically, the incorporation of 1 wt.% TiO_2_ NPs into the NPSE matrix led to a dramatic improvement in formic acid selectivity (FE_HCOOH_ of up to 98.6%) with a high single‐pass product concentration of 17.4 wt.% under electrolyte‐free conditions. Mechanistic analyses revealed that TiO_2_ not only increases the IEC and decreases ohmic resistance but also facilitates the transport of formate anions (HCOO^−^). This improved HCOO^−^ mobility was shown to suppress back‐diffusion of formic acid and stabilize the local cathodic pH, which collectively enhanced CO_2_RR selectivity. These findings highlight the importance of structural and compositional tuning in designing solid electrolytes for efficient CO_2_RR. The NPSE‐TiO_2_ composite presents a promising platform for scalable, selective, and energy‐efficient CO_2_‐to‐formic acid conversion, offering insights for future development of advanced ion‐conductive media. Future work should explore alternative ionomers and nanomaterials for SSE to further enhance ion transport and electrochemical performance.

## Conflict of Interest

The authors declare no conflict of interest.

## Supporting information



Supporting Information

## Data Availability

The data that support the findings of this study are available from the corresponding author upon reasonable request.

## References

[1] Z. Liu , Z. Deng , S. J. Davis , P. Ciais , Nat. Rev. Earth Environ. 2024, 5, 253. 10.1038/s43017-023-00406-z PMC10010646 37065615

[2] P. X. Lei , S. Q. Liu , Q. R. Wen , J. Y. Wu , S. Wu , X. Wei , R. Feng , X. Z. Fu , J. L. Luo , Angew. Chem., Int. Ed. 2025, 64, 202415726. 10.1002/anie.202415726 39240581

[3] L. P. Chi , Y. C. Zhang , Z. Z. Niu , X. L. Zhang , Y. C. Li , T. Y. Zhang , S. P. Sun , P. G. Lu , K. B. Tang , M. R. Gao , Angew. Chem., Int. Ed. 2025, 64, 202503539. 10.1002/anie.202503539 40229217

[4] J. Y. Li , J. R. Huang , Z. H. Zhao , H. L. Zhu , P. Q. Liao , X. M. Chen , Angew. Chem., Int. Ed. 2025, 64, 202511132. 10.1002/anie.202511132 40586154

[5] Y. Liu , Z. Wei , X. Su , X. Shi , L. Liu , T. Wang , X. Xu , M. Zhao , Y. Zhai , H. B. Yang , B. Liu , Adv. Funct. Mater. 2024, 35, 2403547.

[6] Z. Wei , J. Ding , Z. Wang , A. Wang , L. Zhang , Y. Liu , Y. Guo , X. Yang , Y. Zhai , B. Liu , Angew. Chem., Int. Ed. 2024, 63, 202402070. 10.1002/anie.202402070 38664999

[7] R. E. Siegel , M. Aceves , L. A. Berben , ACS Energy Lett. 2024, 9, 2896.

[8] S.‐Q. Liu , M.‐R. Gao , S. Wu , R. Feng , Y. Wang , L. Cui , Y. Guo , X.‐Z. Fu , J.‐L. Luo , Energy Environ. Sci. 2023, 16, 5305.

[9] Y. Liu , Z. Jiang , C. Huang , S. Jeong , A. L. Coughlin , S. Zhang , Y. Liu , X. Ye , Nano Lett. 2023, 23, 5911. 37339508 10.1021/acs.nanolett.3c00703

[10] L. P. Chi , Z. Z. Niu , Y. C. Zhang , X. L. Zhang , J. Liao , Z. Z. Wu , P. C. Yu , M. H. Fan , K. B. Tang , M. R. Gao , Proc. Natl. Acad. Sci. USA 2023, 120, 2312876120. 10.1073/pnas.2312876120 PMC10742388 38085783

[11] K. Fernández‐Caso , G. Díaz‐Sainz , M. Alvarez‐Guerra , A. Irabien , ACS Energy Lett. 2023, 8, 1992.

[12] D. Tan , W. Lee , Y. E. Kim , Y. N. Ko , M. H. Youn , Y. E. Jeon , J. Hong , J. E. Park , J. Seo , S. K. Jeong , Y. Choi , H. Choi , H. Y. Kim , K. T. Park , ACS Appl. Mater. Interfaces 2022, 14, 28890. 35714281 10.1021/acsami.2c05596

[13] L. P. Chi , Z. Z. Niu , X. L. Zhang , P. P. Yang , J. Liao , F. Y. Gao , Z. Z. Wu , K. B. Tang , M. R. Gao , Nat. Commun. 2021, 12, 5835. 34611149 10.1038/s41467-021-26124-y PMC8492718

[14] W. Lee , Y. E. Kim , M. H. Youn , S. K. Jeong , K. T. Park , Angew. Chem., Int. Ed. 2018, 57, 6883. 10.1002/anie.201803501 29660257

[15] S. Y. Choi , S. K. Jeong , H. J. Kim , I.‐H. Baek , K. T. Park , ACS Sustain. Chem. Eng. 2016, 4, 1311.

[16] D.‐S. Huang , Y. Wang , Y. Tang , J.‐R. Huang , P.‐X. Li , C.‐P. Liang , Z.‐H. Zhao , P.‐Q. Liao , X.‐M. Chen , Natl. Sci. Rev. 2025, 12, nwaf329. 41040489 10.1093/nsr/nwaf329 PMC12485609

[17] P. Zhu , H. Wang , Nat. Catal. 2021, 4, 943.

[18] Y. Kang , T. Kim , K. Y. Jung , K. T. Park , Catalysts 2023, 13, 955.

[19] H. Yang , J. J. Kaczur , S. D. Sajjad , R. I. Masel , J. CO2 Util. 2017, 20, 208.

[20] L. Fan , C. Xia , P. Zhu , Y. Lu , H. Wang , Nat. Commun. 2020, 11, 3633. 32686669 10.1038/s41467-020-17403-1 PMC7371694

[21] T. Zheng , C. Liu , C. Guo , M. Zhang , X. Li , Q. Jiang , W. Xue , H. Li , A. Li , C. W. Pao , J. Xiao , C. Xia , J. Zeng , Nat. Nanotechnol. 2021, 16, 1386. 34531557 10.1038/s41565-021-00974-5

[22] L. Lin , X. He , X. G. Zhang , W. Ma , B. Zhang , D. Wei , S. Xie , Q. Zhang , X. Yi , Y. Wang , Angew. Chem., Int. Ed. 2023, 62, 202214959. 10.1002/anie.202214959 36307930

[23] R. Nankya , Y. Xu , A. Elgazzar , P. Zhu , T. U. Wi , C. Qiu , Y. Feng , F. Che , H. Wang , Angew. Chem., Int. Ed. 2024, 63, 202403671. 10.1002/anie.202403671 38887161

[24] S. Guan , Z.‐N. Chen , L.‐Q. Liu , Y.‐K. Li , C.‐Y. Lan , J.‐Z. Wang , P.‐F. Yin , J. Yang , H. Liu , X.‐W. Du , C. Dong , ACS Appl. Energy Mater. 2024, 7, 3201.

[25] C. Zhang , X. Hao , J. Wang , X. Ding , Y. Zhong , Y. Jiang , M. C. Wu , R. Long , W. Gong , C. Liang , W. Cai , J. Low , Y. Xiong , Angew. Chem., Int. Ed. 2024, 63, 202317628. 10.1002/anie.202317628 38305482

[26] Y. Kang , Y. Kim , Y. Doh , J. Lee , J. Kim , K. T. Park , Angew. Chem., Int. Ed. 2025, 137, 202504380. 10.1002/anie.202504380 40289024

[27] Z. H. Zhao , J. R. Huang , D. S. Huang , H. L. Zhu , P. Q. Liao , X. M. Chen , J. Am. Chem. Soc. 2024, 146, 14349. 38742424 10.1021/jacs.4c04841

[28] T.‐U. Wi , Z. H. Levell , S. Hao , A. Elgazzar , P. Zhu , Y. Feng , F.‐Y. Chen , W. P. Lam , M. Shakouri , Y. Liu , H. Wang , ACS Energy Lett. 2025, 10, 822.

[29] Y. Xia , P. Zhu , Y. Yang , C. Qiu , H. Wang , ACS Catal. 2025, 15, 4560.

[30] M. D. Zhang , J. R. Huang , C. P. Liang , X. M. Chen , P. Q. Liao , J. Am. Chem. Soc. 2024, 146, 31034. 39495344 10.1021/jacs.4c10675

[31] Y. C. Liu , J. R. Huang , H. L. Zhu , X. F. Qiu , C. Yu , X. M. Chen , P. Q. Liao , Nat. Nanotechnol. 2025, 20, 907. 40247139 10.1038/s41565-025-01914-3

[32] L. Hu , J. A. Wrubel , C. M. Baez‐Cotto , F. Intia , J. H. Park , A. J. Kropf , N. Kariuki , Z. Huang , A. Farghaly , L. Amichi , P. Saha , L. Tao , D. A. Cullen , D. J. Myers , M. S. Ferrandon , K. C. Neyerlin , Nat. Commun. 2023, 14, 7605. 37989737 10.1038/s41467-023-43409-6 PMC10663610

[33] L. H. Cherniack , K. U. Hansen , Z. Li , A. K. Taylor , K. C. Neyerlin , F. Jiao , ACS Energy Lett. 2025, 10, 1508.

[34] D. Joseph , J. Büsselmann , C. Harms , D. Henkensmeier , M. J. Larsen , A. Dyck , J. H. Jang , H.‐J. Kim , S. W. Nam , J. Membr. Sci. 2016, 520, 723.

[35] M. Jung , W. Lee , N. Nambi Krishnan , S. Kim , G. Gupta , L. Komsiyska , C. Harms , Y. Kwon , D. Henkensmeier , Appl. Surf. Sci. 2018, 450, 301.

[36] H. Liu , Q. She , J. Membr. Sci. 2022, 650, 120398.

[37] M. Cho , S. Han , J. Jang , S. Choi , R. Kwak , B. Kim , J. Membr. Sci. 2025, 717, 123620.

[38] E. Moukheiber , G. De Moor , L. Flandin , C. Bas , J. Membr. Sci. 2012, 389, 294.

[39] Z. Bi , K. Li , C. Jiang , J. Zhang , S. Ma , C. Alberto , M. Sun , Y. Bu , M. Barati , S. Ren , ACS Omega 2022, 7, 21225. 35935296 10.1021/acsomega.2c02252 PMC9347970

[40] X. Song , Y. Wang , D. Song , C. An , J. Wang , Nanomater. Nanotechnol. 2016, 6, 23.

[41] D. Fein , J. Catal. 2002, 210, 241.

[42] B. Rutjens , K. von Foerster , B. Schmid , H. Weinrich , S. Sanz , H. Tempel , R.‐A. Eichel , Ind. Eng. Chem. Res. 2024, 63, 3986.

[43] H. Yang , J. J. Kaczur , S. D. Sajjad , R. I. Masel , J. CO2 Util. 2020, 42, 101349.

