# Supplementary material for: Highly Selective CO2 Reduction to Pure Formic Acid Using a Nafion‐TiO2 Composite Porous Solid Electrolyte
Source: Adv Sci (Weinh). 2025 Oct 13;13(2):e15967. doi: 10.1002/advs.202515967 (PMC12786315; doi:10.1002/advs.202515967)
Supplement: Supplementary file 1 — Supporting Information [file ADVS-13-e15967-s001.pdf]

Supporting Information

Highly selective CO_2 reduction to Pure Formic Acid using a Nafion-TiO_2 Composite Porous Solid Electrolyte

*Yeomin Kang, Kooyoung Jung, Jusung Kim, Ki Tae Park**

[a]Department of Chemical Engineering, Konkuk University, 120 Neungdong-ro, Gwangjin-gu, Seoul 05029, Republic of Korea

Corresponding Author

E-mail addresses: kpark@konkuk.ac.kr

Materials

Chemicals for the preparation of NPSE and electrode, and for the electrochemical measurements were purchased from commercial suppliers and used without any further purification, including Nafion® D2021 dispersion (20% w/w in water and 1-propanol, Alfa Aesar), Nickel foam (eq-bcnf-16m, purity > 99.99%, porosity ≥ 95%, pore diameter ~0.25 mm, MTI), polydimethylsiloxane (20 Shore A, Xinus), isopropyl alcohol (HPLC grade, J.T. Baker), titanium dioxide nanoparticles (>99%, ~150 nm, rutile mix, Sigma-Aldrich), sulfuric acid (98%,Sigma-Aldrich), hydrogen peroxide (30-35.5%, Samchun), Bismuth oxide (Bi_2O_3) nanopowder (99.9%, 80-200 nm, Thermo scientific), Iridium (99.99%, Alfa Aesar), Ru(99.98%, Alfa Aesar), Nafion® D520 dispersion (5 wt% Nafion solution, Dupont). Sustainion® XA-9 dispersion (5% ethanol, Dioxide materials), Nafion® 115 membrane (DuPont), FAA-3-30 membrane (Fumatech), Ti felt (Porosity 75%, thickness 0.35 mm, Bekaert), and carbon paper (39BB, Sigracet), NaCl salt (>99.9%, Hanlab), phenolphtalein (Sigma-Aldrich), NaOH solution (0.1 M, Samchun). Deionized water was obtained by New Human Power II water purification system.

Preparation of Nafion-based porous solid electrolyte (NPSE)

The NPSEs were prepared via a metal foam leaching method. Ni foam was cut to 4.84 cm^2 (2.2 cm x 2.2 cm) pieces. The Ni foam was then pressed to a porosity of ~90% (for NPSE with 75% porosity) and cleaned with DI water and ethanol. NPSE thicknesses were controlled by starting with Ni foam of different thicknesses being pressed to the same porosity of ~90%. The lower density NPSEs with 60% porosity were prepared via the same metal leaching method, only the Ni foam was compressed to a porosity of ~80% before the subsequent steps. Nafion® D2021 dispersion was pipetted into pre-made square PDMS molds. Ni foam pieces were immediately

inserted and rested for 2 min. Nafion dispersion solvents were evaporated at a temperature of 50 °C on a heating plate for 30 minutes to form a co-Ni foam-Nafion structure, then removed from the mold and annealed at a temperature of 135 °C in a vacuum oven depressurized to -0.04 MPa for 60 min. [1] The annealed co-Ni foam-Nafion structure was then treated with a mixture of equal parts DI water, concentrated sulfuric acid, and 30% hydrogen peroxide solution. The structure was left overnight to completely remove the Ni foam scaffolding. The resulting NPSE was washed several times in DI water and pretreated 1 M H_2SO_4 to convert to H^+-form before use. A step-by-step procedure has been provided in Figure S1.

A summary of the NPSE sample names and corresponding properties are provided in Table S1. Thickness was measured by micrometer and measurement of porosity is detailed in the SI section "Porosity measurement of NPSE samples"

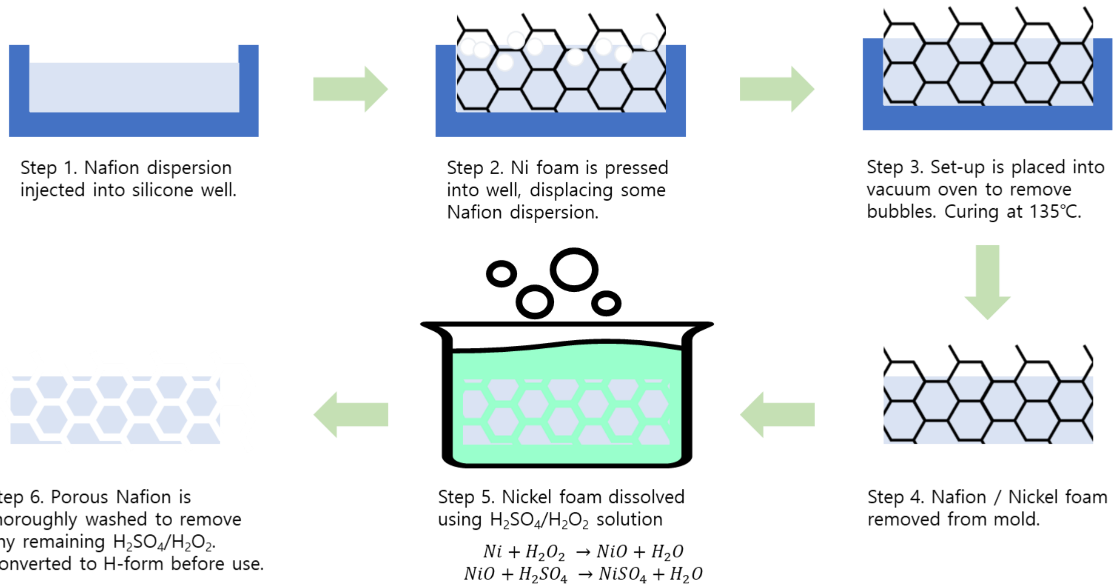

Figure S1. Schematic preparation procedure of NPSE via a metal foam leaching method.

SEM-EDS imaging for NPSE samples

NPSE samples in Na^+-form were washed to remove excess NaCl and dried overnight in a convection oven set to 70 °C. After platinum coating, field emission scanning electron microscopy (FE-SEM) images and energy-dispersive X-ray spectroscopy (EDS) were observed using ZEISS Auriga at an accelerating voltage of 30 kV.

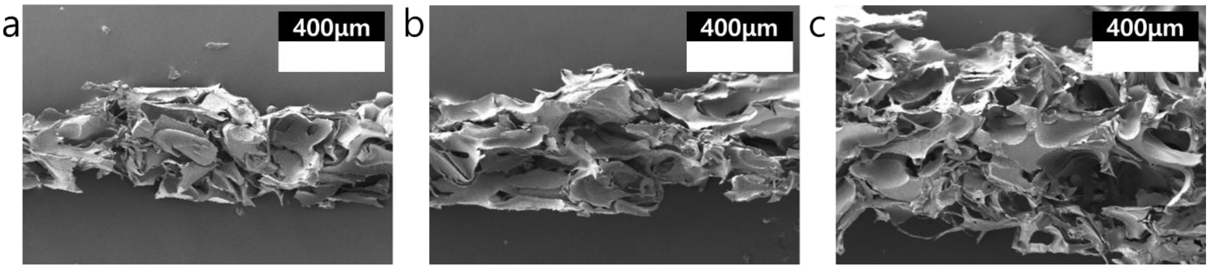

Figure S2. Cross-sectional SEM images of NPSE samples with thicknesses of 400 μm (a), 600 μm (b) and 1000 μm (c), showcasing the open-pore structure of NPSEs.

Porosity measurements of NPSE samples

Porosity of NPSE samples was calculated using Equation (1)

$$\Phi = \left(1 - \frac{\frac{m_{Dry}}{\rho_{Dry}} + \frac{m_{H2O}}{\rho_{H2O}}}{V_T}\right) \times 100(\%) \quad (1)$$

Where m denotes mass, ρ represents specific mass, and V_T is the total volume of NPSE samples including void volume.

As the NPSE is a gel that contains water, water uptake must be taken into consideration to accurately assess porosity. Both m_{Dry} and m_{Wet} were measured to calculate the mass of water contained within the samples. m_{Wet} was measured immediately after excess water was wiped off NPSE samples, while m_{Dry} was measured after drying NPSE samples in a convection oven set to 70 °C overnight. The volume of NPSE samples were controlled by cutting them to 1 cm^2 (1 cm x 1 cm) pieces. The thicknesses were defined by gasket thickness considering compression. All tests were repeated 3 times.

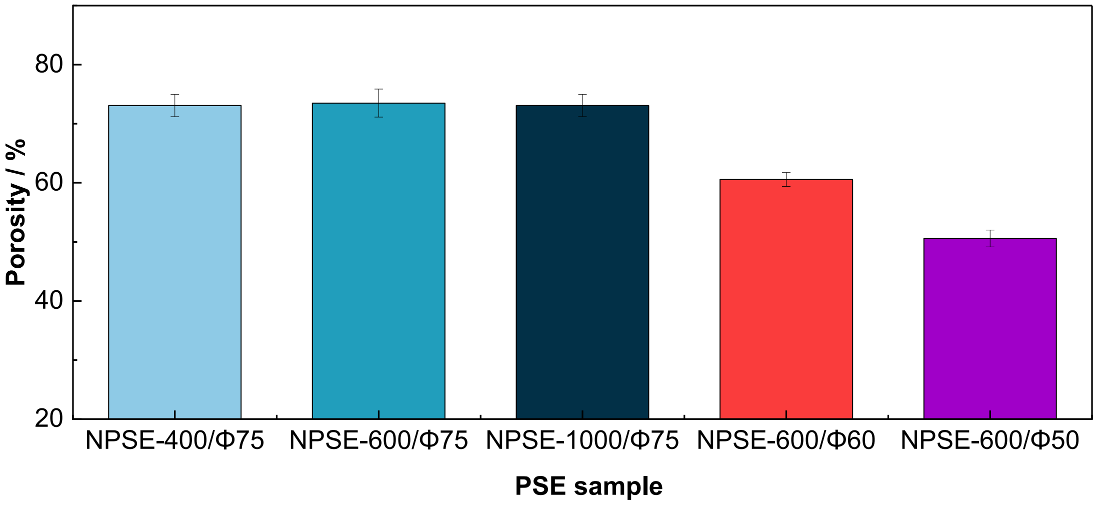

Figure S3. Porosity measurements of prepared NPSE samples.

Table S1. Prepared NPSE samples and their physical properties.

NPSE samples	Thickness	Porosity
NPSE-400/Φ75	413 μm	73.1%
NPSE-600/Φ75	617 μm	73.5%
NPSE-1000/Φ75	1041 μm	73.1%
NPSE-600/Φ60	626 μm	60.6%
NPSE-600/Φ50	615 μm	50.6%

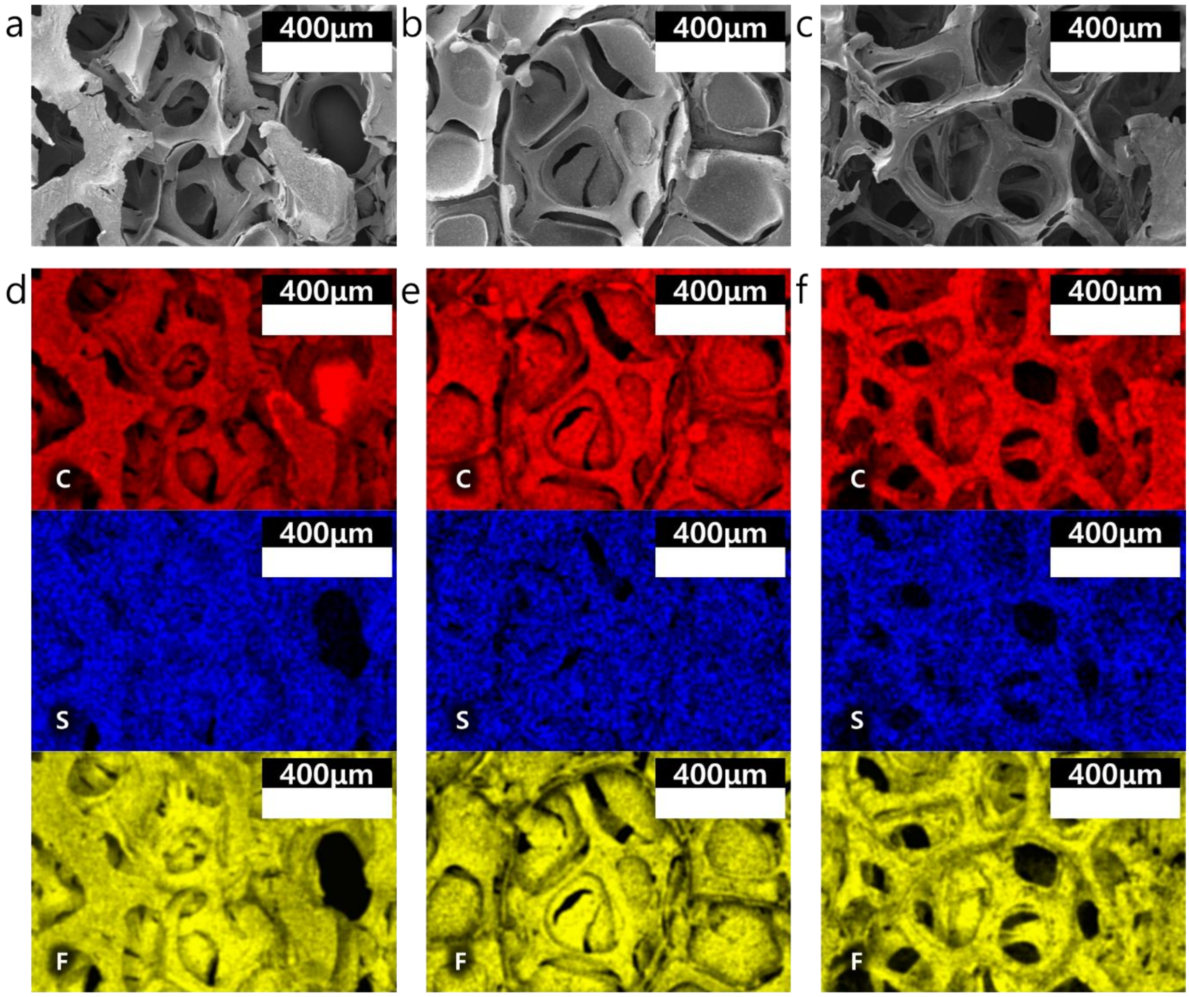

Figure S4. SEM and EDS images of NPSE samples having thicknesses of 400 μm (a, d), 600 μm (b, e) and 1000 μm (c, f).

Table S2. Mass normalized element content according to EDS measurements.

Element	Normalized Mass [%]			
	**NPSE-400/Φ75**	**NPSE-600/Φ75**	**NPSE-1000/Φ75**	**NPSE-600/Φ60**
C	23.10	21.99	21.45	21.95
O	13.01	11.92	12.39	12.52
F	50.69	42.98	49.94	51.94
Na	5.87	10.29	7.36	5.98
S	2.56	2.20	2.42	2.57
Cl	4.78	9.70	5.65	4.33
Ni	0.00	0.91	0.79	0.72
SUM	100.0	100.0	100.0	100.0

EIS measurement of isolated NPSE samples

EIS measurements (1 MHz to 100 Hz at a cell voltage of 0.0 V with an amplitude of 10 mV) were conducted by a custom solid electrolyte EIS jig (Figure S5, S6). Prepared NPSE samples were pretreated in 1 M H_2SO_4 solutions overnight to convert to H^+-form. The samples were then thoroughly washed with DI water to remove excess sulfuric acid and cut into discs of 5.0 mm diameter. PTFE gaskets with thicknesses to achieve 10% compression were inserted alongside NPSE samples to maintain constant contact resistance.

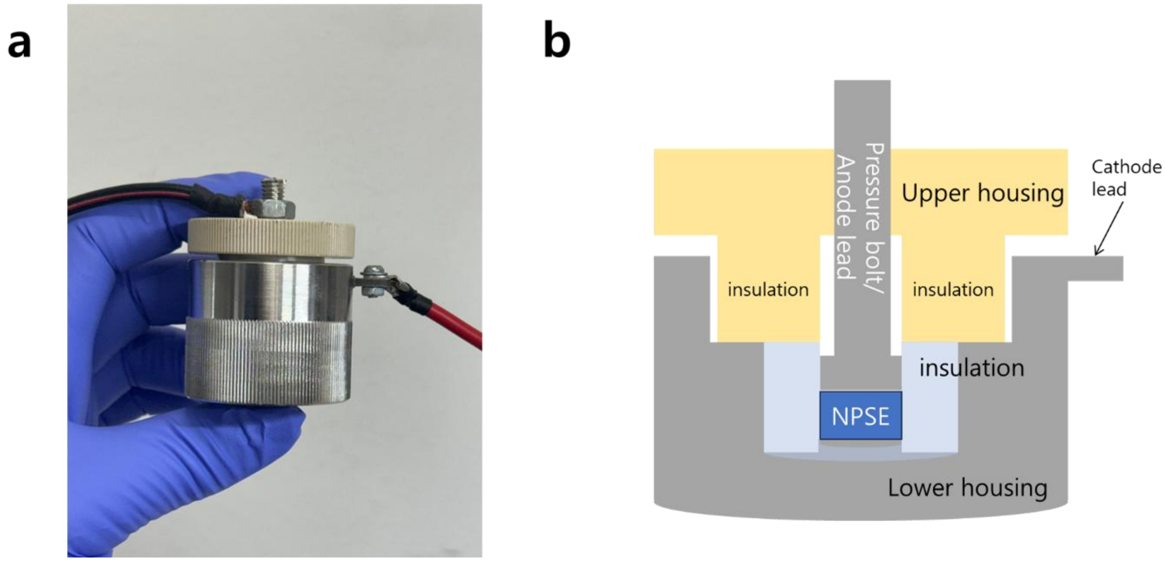

Figure S5. Electrochemical impedance spectroscopy (EIS) measurement cell for isolated solid electrolyte. (a) Photographic image and (b) schematic diagram.

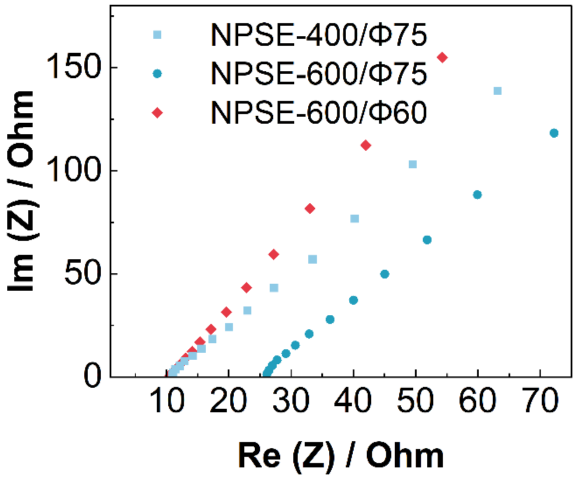

Figure S6. EIS results of isolated NPSEs using the EIS measurement cell. Results for NPSE-600/Φ60 are shown with NPSE-400/Φ75 and NPSE-600/Φ75 for comparison. (1 MHz to 100 Hz, E_{OC}, 10 mV amplitude)

Ion exchange capacity measurements for NPSE samples

Pretreated NPSE samples converted to H^+-form were washed to remove excess sulfuric acid and subsequently dried and weighed to obtain dry mass. The dried NPSE samples were treated with saturated NaCl solution overnight to kick protons bound to NPSE functional groups into solution. Titration was conducted with 0.01 M NaOH using phenolphthalein as the indicator. IEC was calculated using equation (2):

$$ICE = \frac{V_{NaOH}(\mu l) \times C_{NaOH}(M)}{m_{dry}(mg)} \tag{2}$$

Here, *V* denotes the volume of titrant (NaOH), and *C* is the concentration of titrant (0.01 M). All tests were repeated 3 times.

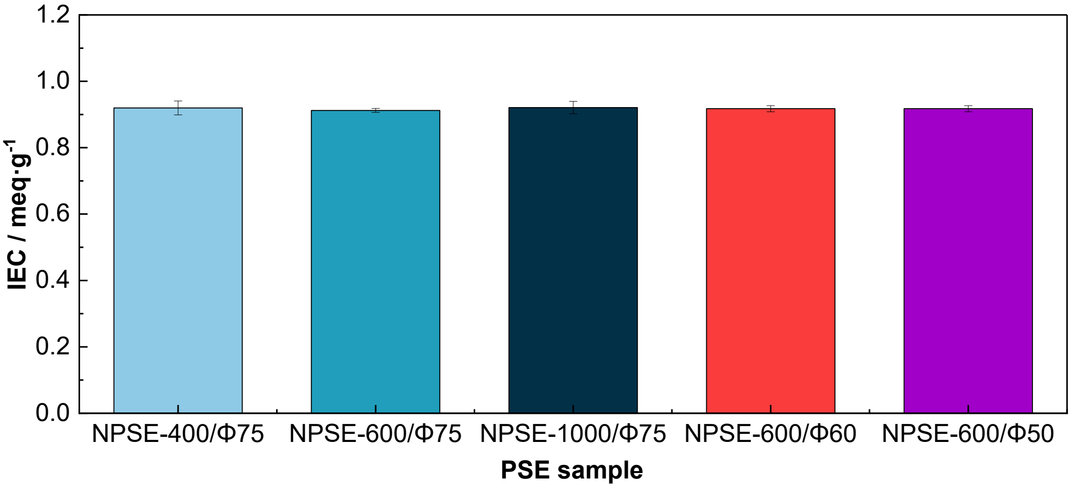

Figure S7. Ion exchange capacity (IEC) measurements of prepared NPSE samples.

Preparation of bismuth oxide cathodes for electrochemical NPSE cell measurements

Bismuth oxide cathodes were fabricated by spray-coating catalyst slurry onto a 36 cm² section (6 cm × 6 cm) of carbon paper, achieving a Bi_2O_3 loading of 2.0 mg/cm². The catalyst ink was produced by blending Bi_2O_3 particles with Sustainion dispersion in isopropyl alcohol, with the mixture undergoing ultrasonication for 120 min. Ultrasonication of the catalyst slurry was maintained during the spray process. The electrodes were then cut to a size of 2 cm × 2 cm and allowed to dry at an ambient temperature for 12 h prior to use.

Electrocatalytic CO_2 reduction experiments

Electrocatalytic CO_2 reduction reaction (CO_2RR) experiments were conducted in an SSE cell with three compartments. CO_2RR performance was assessed using chronopotentiometry (CP) for 20 min at different currents. The anodic and center compartments were separated by a Nafion 115 proton exchange membrane, while the center and cathodic compartments were separated by a FAA-3-30 anion exchange membrane. The anode was a titanium felt coated with Ir-Ru catalyst, and the cathode was a carbon paper GDE coated with Bi_2O_3 catalyst. Current collectors, featuring serpentine-type flow channels, were employed for both electrodes, where the materials used were titanium and nickel for anode and cathode respectively. The GDE electrodes, NPSE, membranes, and current collectors for both electrodes were assembled with gaskets made of PTFE for sealing and were then sandwiched between two end plates (SUS 316) and tightened using fastening bolts (Fig. S8). The DI water anolyte was fed into the cell at a rate of 3 mL/min, while CO_2 gas was introduced into the cathode channel at a flow rate of 50 sccm, while the center compartment products were retrieved via inert N_2 gas with a custom heated humidifier supplying water vapor at 333 K. The cell temperature was maintained at 303 K. Liquid products collected in a tank were examined using high-performance liquid

chromatography (Ultimate 3000, Thermo Scientific) after each CP test. The CO_2RR performance of the NPSE was evaluated based on Faradaic efficiency (FE), partial current density (*j*) and energy efficiency (EE). Quantitative analyses of the gas and liquid products were conducted to investigate FE and partial current density, which were calculated using equations (3) and (4):

$$FE(\%) = \frac{nNF}{Q} \times 100 \tag{3}$$

$$j(mA/cm^2) = FE \times \frac{Q}{t} \times \frac{1}{A} \tag{4}$$

Where, n and N denote the moles of the product and the electrons for the product, respectively, and *F* represents the Faraday constant (96,485.3 C/mol of electrons). Term *A* refers to the geometric area of the electrode (4 cm^2 in this study), while *Q* (C) is the total charge passed during reaction, and *t* is the reaction time.

Energy efficiency was evaluated using equation (5):

$$EE = \frac{E_{theoretical} \times FE}{E_{applied}} \tag{5}$$

Where $E_{theoretical}$ is the theoretical potential difference for oxygen evolution reaction at the anode (1.23 V vs RHE)[2] and CO_2 reduction to formic acid at the cathode (-0.25 V vs RHE)[3]. FE represents the calculated Faradaic efficiency, and $E_{applied}$ is the average voltage of the cell during CP measurements.

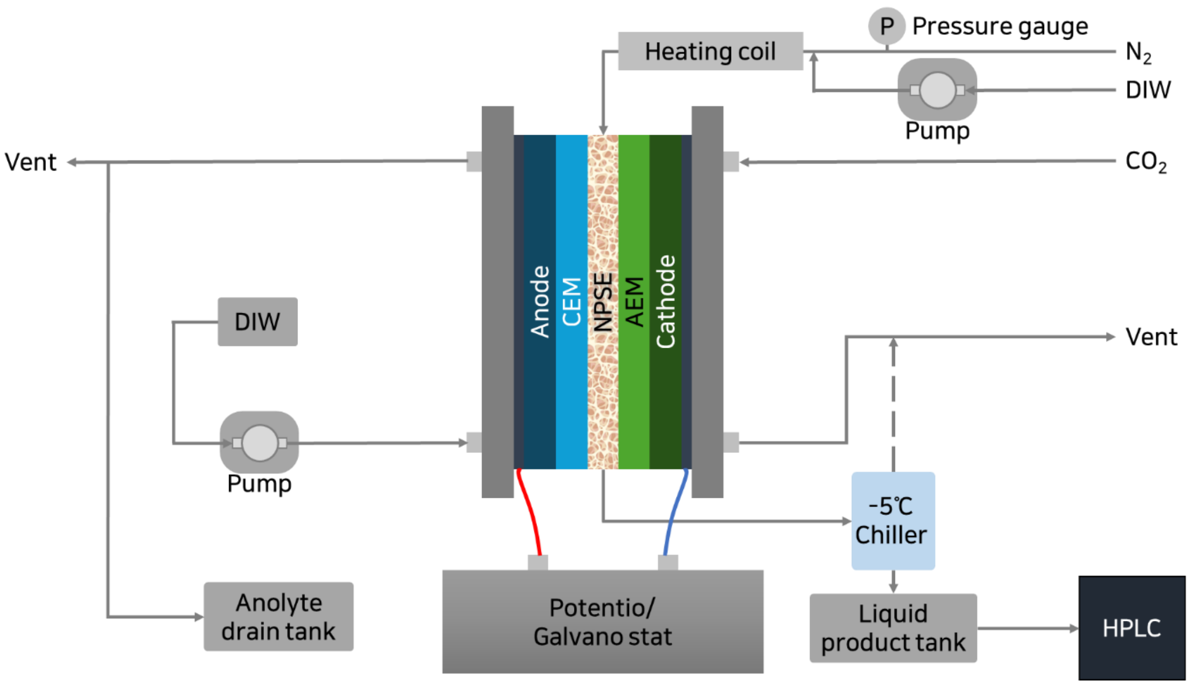

Figure S8. Experimental setup and construction of NPSE-cell for CO_2RR performance test.

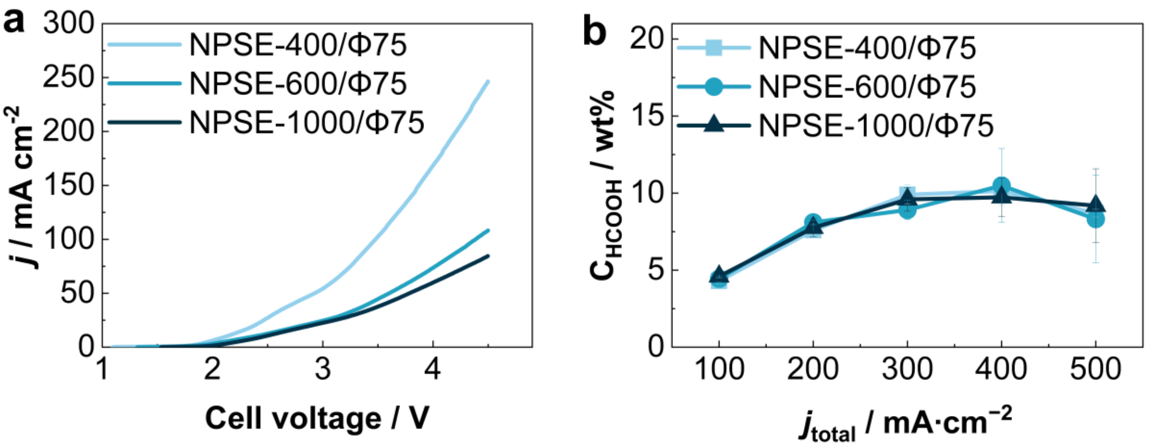

Figure S9. (a) Linear sweep voltammetry (LSV) results and (b) produced formic acid concentration depending on NPSE thickness.

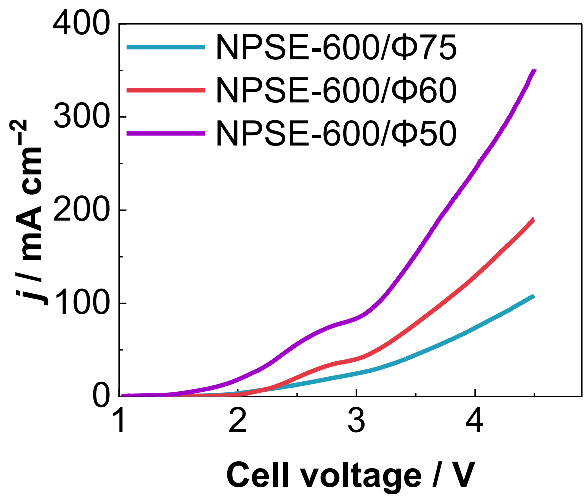

Figure S10. LSV curves for NPSE with different porosities.

Preparation of Nafion-TiO_2 composite porous solid electrolyte (NPSE-TiO_2)

NPSEs incorporating TiO_2 nanoparticles were by first dispersing TiO_2 nanoparticles into isopropyl alcohol to make a 2 mg/ml slurry. The slurries were sonicated for 30 min to form a uniform dispersion. The TiO_2 slurry was sonicated with the Nafion D2021 dispersion at different ratios to match the target content (Table S3). The mixture was then used to prepare NPSE samples following the above procedure.

Table S3. Dispersion mixture ratio recipes

**Dispersion**	**TiO_2 slurry**	**Nafion D2021**	**Nafion** (Dry weight)	**TiO_2** (Dry weight)
TiO_2 slurry	20 ml	-	-	40 mg
Nafion D2021	-	5.0 ml	1000 mg	-
D-$TiO_2$0.5%	2.5 ml	5.0 ml	1000 mg	5 mg
D-$TiO_2$1.0%	5.0 ml	5.0 ml	1000 mg	10 mg
D-$TiO_2$2.0%	10.0 ml	5.0 ml	1000 mg	20 mg

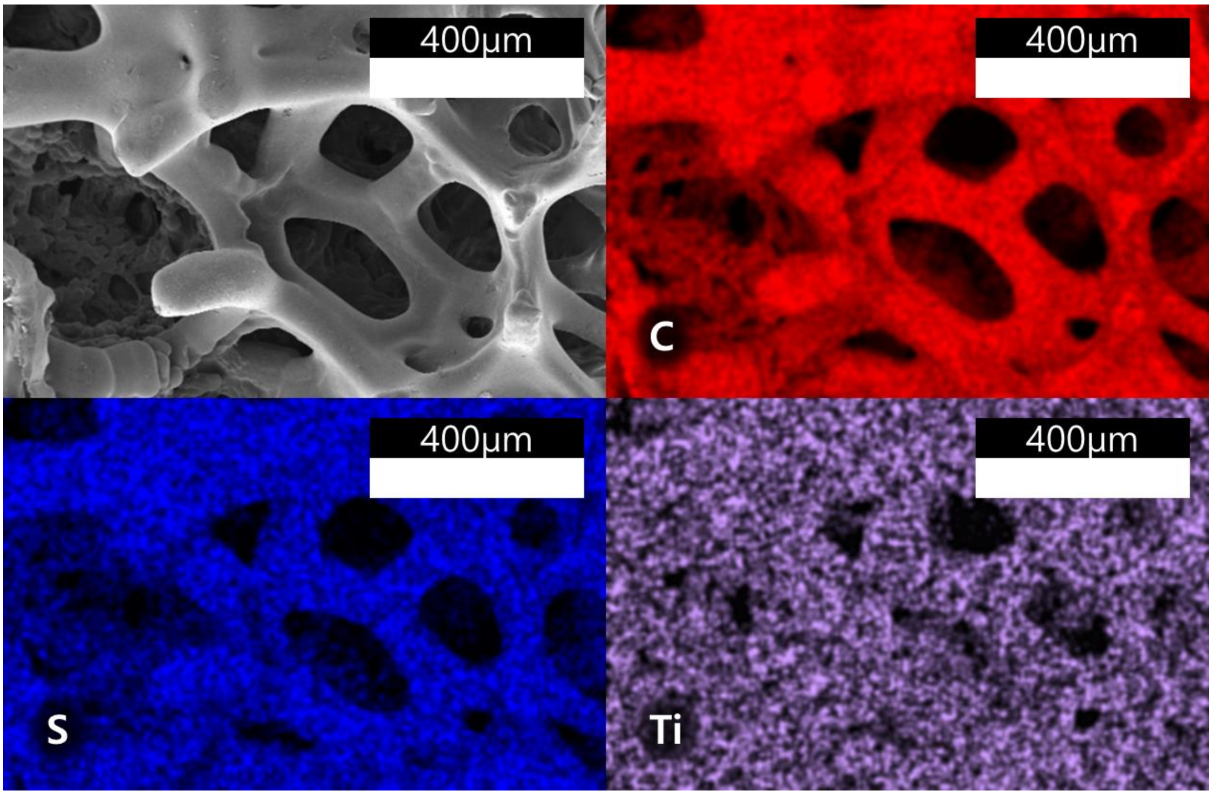

Figure S11. FE-SEM and EDS mapping (C, S, Ti) images of NPSE-600/Φ60/TiO_2 0.5% surface.

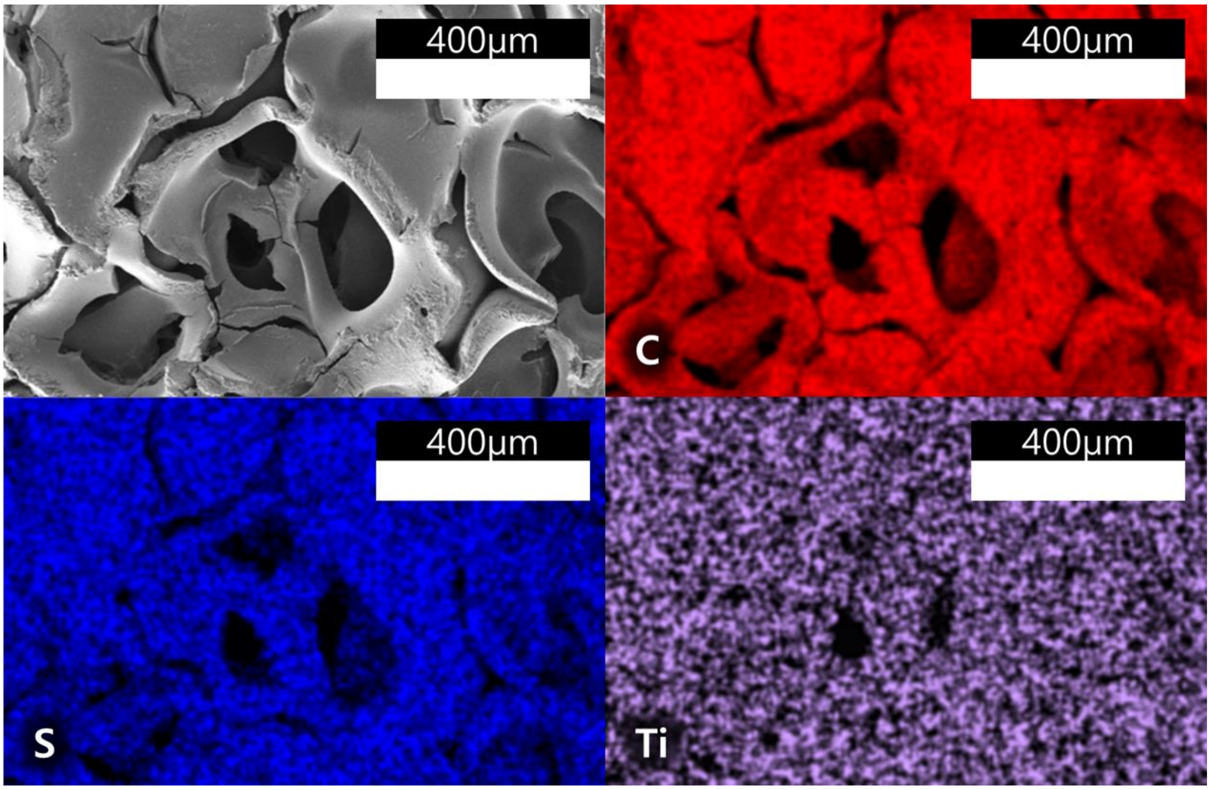

Figure S12. FE-SEM and EDS mapping (C, S, Ti) images of NPSE-600/Φ60/$TiO_2$2% surface.

Table S4. Mass normalized element content according to EDS measurements. Due to the porous nature of NPSEs, observed Ti content diverged from that of the dispersion formula.

Element	Normalized Mass [%]		
	NPSE-600/Φ60/TiO_2 0.5%	NPSE-600/Φ60/TiO_2 1%	NPSE-600/Φ60/TiO_2 2%
**C**	23.06	21.93	20.90
**O**	16.04	14.51	15.07
**F**	50.51	51.65	51.32
**Na**	2.12	3.04	2.55
**S**	2.16	3.05	3.14
**Cl**	0.00	0.00	0.00
**Ni**	0.00	0.00	0.00
**Ti**	6.11	6.91	8.01
**SUM**	100.0	100.0	100.0

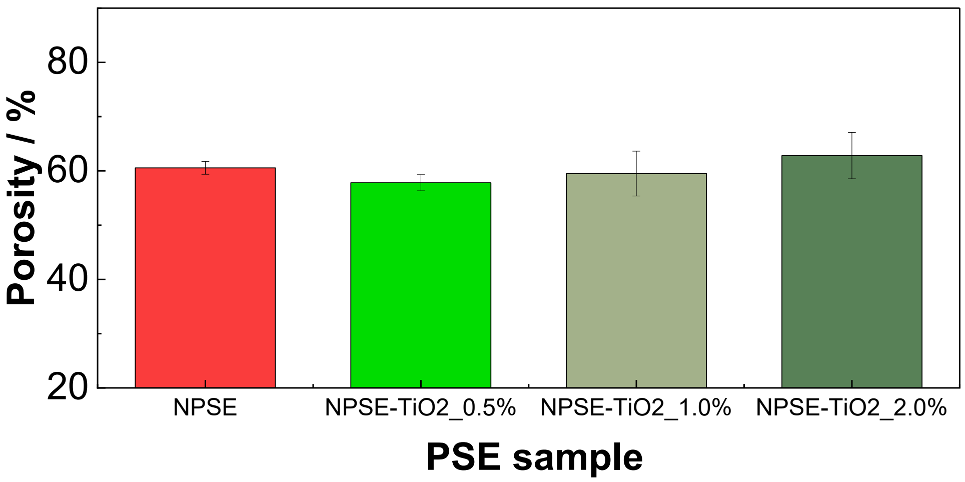

Figure S13. Measured porosity of NPSE samples according to TiO_2 content. The measurement procedure was identical to that of NPSE without TiO_2.

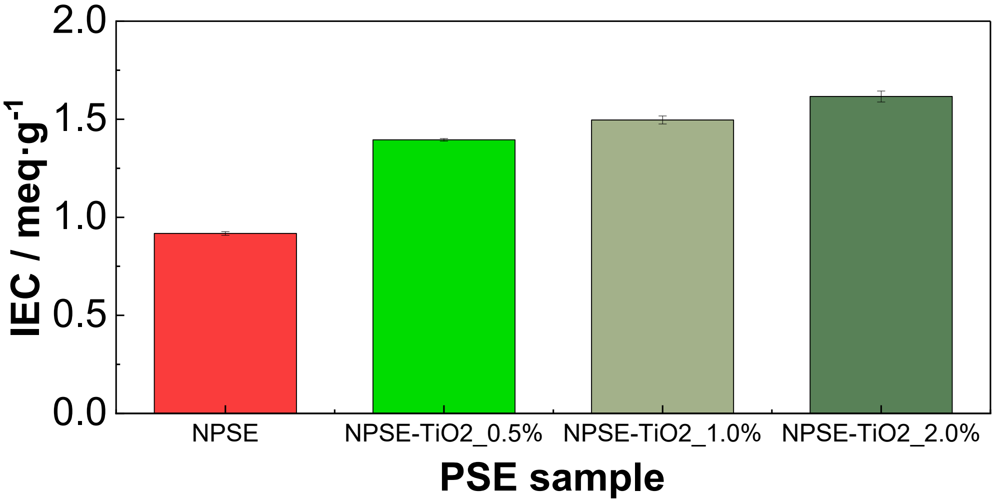

Figure S14. Ion exchange capacity measurements of prepared NPSE samples. The measurement procedure was identical to that of NPSE without TiO_2.

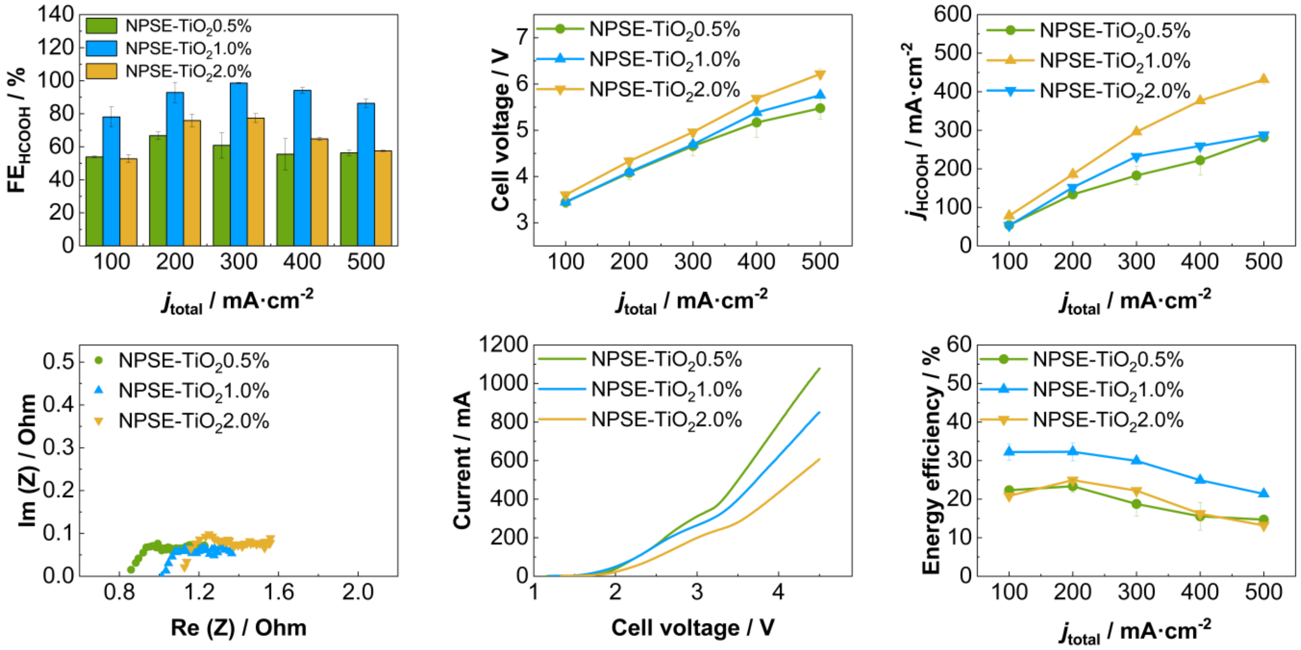

Figure S15. CO_2RR performance depending on TiO_2 content. (a) FE, (b) Cell voltage, (c) partial current density, (d) Nyquist impedance plots (1 MHz to 10 Hz, 3.8 V_{cell}, 30 mV amplitude), (e) LSV curves (OCV to 4.5 V, 50 mV s^{-1}), and (f) Energy efficiency.

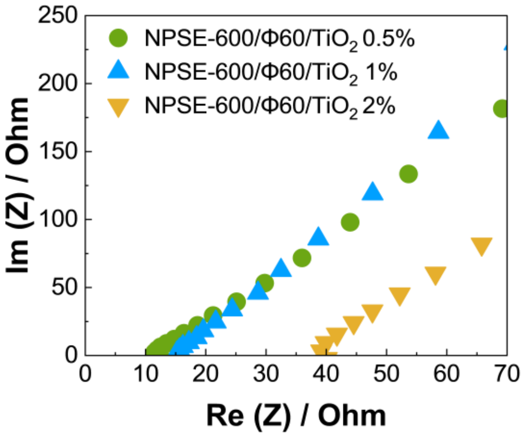

Figure S16. Nyquist impedance plots (1 MHz to 100 Hz, E_{OC}, 10 mV amplitude) for isolated NPSE samples according to TiO_2 content using the solid electrolyte analysis jig (See Figure S5).

Casting Nafion membranes containing/not containing TiO_2

Dense NM-$TiO_2$1% membranes were drop-cast onto PDMS sheets using the dispersion D-$TiO_2$1%. The NM membranes were made in a similar fashion but Nafion D2021 was diluted with isopropyl alcohol to match the viscosity of D-$TiO_2$1%. After drying the dispersions in ambient conditions overnight, the membranes were annealed in a 135 °C oven for 1hr. The resulting samples were then washed and kept in DI water until use.

TiO_2 membrane diffusion experiments

Formate diffusion experiments were carried out in a custom H-type cell set-up. After clamping the cast membranes into place, the cell was checked for leakage before being filled. The receiving compartment was filled with 30ml of DI water, while the donating compartment was filled with 30 ml of 0.5 M HCOOK solution. Formate concentrations of the receiving compartment were measured by high-performance liquid chromatography (1260 Infinity II, Agilent) every 30 minutes.

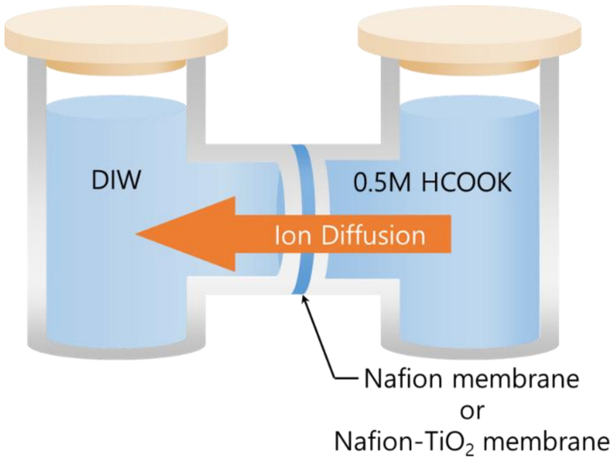

Figure S17. Schematic of diffusion cell setup. Either a pure Nafion membrane or a co-Nafion-TiO_2 membrane was used to separate the two compartments to assess the ion conducting properties.

Formate-TiO_2 adsorption experiments

A solution of dilute potassium formate (HCOOK) was prepared, and concentration was measured to be 85μM. To 5mL of this solution was added 200mg of TiO_2 nanoparticles to form a dispersion. The original solution and TiO_2 added dispersion were subjected to sonication for 30 minutes. After centrifugal separation, the supernatant concentration was measured via liquid chromatography.

Adsorption site density was measured using equation (6)

$$N_S = \frac{Adsorption\ capcity(\mu mol/g)}{BET(m^2/g)} \tag{6}$$

Where BET is BET surface area. BET values used for calculation were taken from the material provider's specification sheet.

Table S5. Calculated values for adsorption capacity and adsorption site density derived from bulk adsorption results.

**Adsorption capacity** μmol / g	**Adsorption site density** μmol / m^2
352.90	5.43

References

[1] D. Joseph, J. Büsselmann, C. Harms, D. Henkensmeier, M. J. Larsen, A. Dyck, J. H. Jang, H.-J. Kim, S. W. Nam, *Journal of Membrane Science* **2016**, *520*, 723-730.

[2] K. Zeng, D. Zhang, *Progress in Energy and Combustion Science* **2010**, *36*, 307-326.

[3] J. W. Vickers, D. Alfonso, D. R. Kauffman, *Energy Technology* **2017**, *5*, 775-795.
